# Training emergency services’ dispatchers to recognise stroke: an interrupted time-series analysis

**DOI:** 10.1186/1472-6963-13-318

**Published:** 2013-08-15

**Authors:** Caroline L Watkins, Michael J Leathley, Stephanie P Jones, Gary A Ford, Tom Quinn, Chris J Sutton

**Affiliations:** 1 Clinical Practice Research Unit, School of Health, University of Central Lancashire, Preston, UK; 2 Level 6 Leazes Wing, Royal Victoria Infirmary, Newcastle, UK; 3 Faculty of Health and Medical Sciences, Duke of Kent Building, University of Surrey, Guildford, Surrey, UK

**Keywords:** Emergency medical services, Emergency medical dispatchers, Stroke, Recognition training

## Abstract

**Background:**

Stroke is a time-dependent medical emergency in which early presentation to specialist care reduces death and dependency. Up to 70% of all stroke patients obtain first medical contact from the Emergency Medical Services (EMS). Identifying ‘true stroke’ from an EMS call is challenging, with over 50% of strokes being misclassified. The aim of this study was to evaluate the impact of the training package on the recognition of stroke by Emergency Medical Dispatchers (EMDs).

**Methods:**

This study took place in an ambulance service and a hospital in England using an interrupted time-series design. Suspected stroke patients were identified in one week blocks, every three weeks over an 18 month period, during which time the training was implemented. Patients were included if they had a diagnosis of stroke (EMS or hospital). The effect of the intervention on the accuracy of dispatch diagnosis was investigated using binomial (grouped) logistic regression.

**Results:**

In the Pre-implementation period EMDs correctly identified 63% of stroke patients; this increased to 80% Post-implementation. This change was significant (p=0.003), reflecting an improvement in identifying stroke patients relative to the Pre-implementation period both the During-implementation (OR=4.10 [95% CI 1.58 to 10.66]) and Post-implementation (OR=2.30 [95% CI 1.07 to 4.92]) periods. For patients with a final diagnosis of stroke who had been dispatched as stroke there was a marginally non-significant 2.8 minutes (95% CI −0.2 to 5.9 minutes, p=0.068) reduction between Pre- and Post-implementation periods from call to arrival of the ambulance at scene.

**Conclusions:**

This is the first study to develop, implement and evaluate the impact of a training package for EMDs with the aim of improving the recognition of stroke. Training led to a significant increase in the proportion of stroke patients dispatched as such by EMDs; a small reduction in time from call to arrival at scene by the ambulance also appeared likely. The training package has been endorsed by the UK Stroke Forum Education and Training, and is free to access on-line.

## Background

Stroke is a leading cause of mortality and disability worldwide [1] and is increasingly recognised as a time-dependent medical emergency in which early presentation to specialist care reduces death and dependency [2].

Up to 70% of all stroke patients obtain first medical contact from the Emergency Medical Services (EMS) [2-4]. Calls to the EMS are triaged using Advanced Medical Priority Dispatch System (AMPDS) [5], a system also used widely in Europe and North America. Emergency Medical Dispatchers (EMDs) use this system to categorise ambulance response and decide on the level of medical care sent. If the EMD suspects a time critical condition such as stroke, an ambulance can be dispatched as a high priority (category A, currently up to a 19 minute response). In the United Kingdom the categories for response prioritisation are pre-determined by the Department of Health.

Identifying ‘true stroke’ from an EMS call is challenging and unlike many other health care systems, EMDs in the UK have no specialist medical training. Although AMPDS is effective at ruling out acute stroke in people with other conditions, it is poor at correctly identifying acute stroke, with over 50% of strokes being misclassified [6-8]. It is important that stroke is recognised at the earliest opportunity to ensure that an ambulance is dispatched with an appropriate level of priority, thereby facilitating early presentation and rapid specialist treatment such as thrombolysis, where the benefits are highly time dependent.

One way of facilitating rapid EMS transport to hospital, thereby improving the chance for early presentation is through enhancing communication between EMDs and the general public. Within this programme of research, we have previously explored the interaction between EMDs and the public during emergency calls for stroke, in order to inform the content of stroke-specific, on-line training for EMDs; this included exploring callers’ experiences [9] and identifying the key words used by the public to describe and that are indicative of stroke [10].

The aim of this study was to evaluate the impact of this newly developed stroke-specific, on-line training package on the recognition of stroke by EMDs, and on the impact of stroke recognition on the time between the call to EMS and stroke patients reaching hospital.

## Methods

### Design

Interrupted time-series.

### Setting

An ambulance service and a hospital in the North West of England. The ambulance service in this study receives approximately 50,000 emergency calls each year, 1% of which are for suspected stroke. The hospital provides acute health care to a large urban population of around 330,000.

### Subjects and sampling

Subjects were patients with suspected stroke arriving at hospital by ambulance during an 18 month period (16^th^ March 2009 to 29^th^ August 2010). For every three week period, we identified one week of consecutive patients (arrival at hospital between 0:00 hours on the Monday through to 11:59 hours the following Sunday). Each sampled week was deemed an observation.

Inclusion criteria: patients who had a diagnosis of suspected stroke by the EMS call handler and/or a final diagnosis of stroke or TIA in hospital. Exclusion criteria: patients whose General Practitioner contacted the EMS on their behalf; patients who had a stroke while already an inpatient. The sample was identified through a retrospective chart review of hospital and EMS records. Stroke patients were identified in hospital from a comprehensive stroke register, which is regularly reviewed and updated during a patient’s stay to ensure that only ‘true’ stroke patients stay on the register. Additional patients were identified from hospital by searching the hospital coding system and the Emergency Department (ED) records. Case notes were ordered for any patients recorded as stroke in the coding system or with stroke-like symptoms in the ED records who were not on the register; the case notes were reviewed and the diagnosis checked by an experienced stroke research nurse. For all patients identified in hospital we obtained their EMS data. Independent from the data gathered in hospital, the EMS identified patients who had been dispatched as a stroke. The hospital case notes were obtained for all patients identified through the EMS. A patient was considered to have a final diagnosis of stroke if they were discharged from the ED and the ED records stated stroke or if they were still on the stroke register at the time of discharge from hospital.

Approval for this study was granted by the Patient Information Advisory Group (now the National Information Governance Board for Health and Social Care), the Local Research Ethics Committee and by the Faculty of Health Ethics Committee at the University of Central Lancashire.

### Procedure

Data were recorded from the electronic patient report forms used by EMS staff, and the patient’s hospital case notes using methodological standards for emergency services research [11,12]. Data were extracted by a trained research nurse in accordance with the study protocol, using a standardised data abstraction form. EMS report forms provided data on dispatch code, ambulance diagnosis and the following event times: call made to EMS; ambulance arrived at scene; ambulance arrived at hospital. From the case notes and stroke register we recorded: demographics; side affected by the stroke; limbs affected by stroke; facial weakness; speech problems; conscious level; and final diagnosis. Also recorded were time of admission, and time of triage.

Study data were divided into three periods: Pre-implementation – prior to training the EMS call handlers; During-implementation – during which 69 EMDs (2 trainers and 67 EMDs) completed the training; Post-implementation – following completion of the training.

An on-line training package was developed between 1st December 2008 and 30th June 2009 informed by the results from previous phases of a programme of stroke research. The training package included information about:

•What a stroke is

•Common symptoms of stroke

•Stroke mimics

•The factors that influence the public’s initial decision to contact the EMS at the onset of stroke symptoms

•The importance of effective communication between the EMD and caller

•Who is most likely to contact the EMS for suspected stroke

•How stroke symptoms may be described by the public during calls to the EMS

•Stroke risk factors

The training package took two hours to complete and included a post-training assessment, which consisted of 17 multiple-choice questions.

There were 9, 7 and 10 one-week blocks in the three periods, respectively, giving a total of 26 observations. We dichotomised patient diagnosis as stroke or not stroke at the point of dispatch and for the final diagnosis. Time intervals from call to EMS and other key events (arrival at scene, arrival at hospital) were calculated (as described in Table 1). For each observation (*i*=1, …, 26) we produced the following outcome summary statistics for analysis:

1. Proportion with final diagnosis stroke (*n*_*i*_) dispatched as stroke (*y*_*i*_).

2. Mean time interval between call and ambulance arrival at scene.

3. Mean time interval between call and arrival at hospital.

**Table 1:** Time intervals and their method of calculation

**Time interval**	**Method of calculation**
Call to arrival at scene	Time ambulance arrived at scene minus time call made to EMS
Call to arrival at hospital	Time ambulance arrived at hospital minus time call made to EMS; If time arrived at hospital was missing, time of admission to hospital was used instead; if both these were missing, time of triage was used.

#### Data analysis

Patient demographics, stroke characteristics and diagnosis data are presented overall and for each period. Time series plots are presented to illustrate trends for each outcome.

The effect of the intervention on:

1. accuracy of dispatch diagnosis was investigated using binomial (grouped) logistic regression, with the number of subjects with a dispatch diagnosis of stroke as the numerator and the number of subjects with a final diagnosis of stroke as denominator for each observation;

2. call to arrival at scene and call to arrival at hospital were investigated using linear modelling, with observations weighted by that week’s number admitted with a final diagnosis of stroke.

Analysis was performed using complete cases, i.e. those with data available for both dispatch and final diagnosis. It was suspected that, given the non-contiguous nature of the observation periods, serial autocorrelation would be absent or weak. However, potential autocorrelation (due to the time of the weekly diagnosis rates and clustering [over-dispersion] of the accuracy within observations) was investigated; standard errors would be adjusted for any observed lack of independence or over-dispersion.

Segmented regression models [13] included the intervention factor (pre; during; post: this segments the regression model and allows a ‘jump’ in outcome on transition from one period to the next). An overall linear trend over the period of data collection and an interaction between the intervention factor and the linear trend (to allow the intervention to influence any underlying trend and to allow a gradual impact of the introduction of the intervention) were also included. Where there was no evidence of either an overall trend or difference in trend between periods (p>0.15), the corresponding term was removed; findings from the more parsimonious model are presented. Sensitivity analysis was performed to assess the potential impact of missing dispatch and/or final diagnosis by imputing possible diagnoses, including extreme imputations (dispatch diagnosis as ‘not stroke’ and final diagnosis as ‘stroke’, and vice-versa). For the time intervals, sensitivity analyses were performed by repeating the modelling on geometric rather than arithmetic means for each observation (to reduce the potential influence of outlying times). Analysis was performed using SPSS (versions 19 and 20) and Intercooled Stata (version 11.0). Unless otherwise stated, inferential analyses used a 5% significance level; 95% confidence intervals are presented.

## Results

Over the 26 observation weeks, 464 patients met the study’s inclusion criteria. Sixty-six patients were included due to a final diagnosis of stroke only, 251 patients were included due to having a stroke dispatch code only and 147 patients met both these inclusion criteria. Their median (IQR) age was 75 (62 to 83) years; 241 (51.9%) were female. Data were collected on 174 (mean 19.3 per week), 116 (mean 16.6 per week) and 174 (mean 17.4 per week) patients over the Pre-, During-, and Post-implementation periods respectively. Dispatch data were available for 450 patients and, of these, 398 (88.4%) were dispatched as stroke. A final confirmed diagnosis was recorded for 427 patients, and of these 213 (50.2%) had a final diagnosis of stroke (Table 2).

**Table 2:** Patient characteristics, dispatch category, and final diagnosis for the three periods and overall

	**Pre (N=174)**	**During (N=116)**	**Post (N=174)**	**Overall (N=464)**
Median age years (IQR)	76 (65–82)	74.5 (61–84)	75 (62–83)	75 (62–83)
Female (%)	81/174 (46.6)	63/116 (54.3)	97/174 (55.7)	241/464 (51.9)
Dispatched as stroke (%) (all diagnoses)	134/168 (79.8)	105/111 (94.6)	159/171 (93.0)	398/450 (88.4)
Final Diagnosis of stroke (%)	98/162 (60.5)	53/106 (50.0)	62/159 (39.0)	213/427 (49.9)

Denominator varies by row due to missing data.

Of the 213 patients with a final diagnosis of stroke, dispatch data were available for 199, of whom 147 (73.9%) had been dispatched as stroke. The characteristics of the 199 stroke patients are shown in Table 3.

**Table 3:** Characteristics of patients with final diagnosis of stroke dispatched as stroke

	**Pre (N=92)**	**During (N=48)**	**Post (N=59)**	**Overall (N=199)**
Median age years (IQR)	76 (65 to 83)	75 (64 to 83)	75 (66 to 82)	76 (65 to 83)
Female (%)	38/92 (41.3)	21/48 (43,8)	25/59 (42.4)	84/199 (42.2)
Side affected by stroke:				
No clear lateralisation (%)	23/88 (26.1)	11/46 (23.9)	10/58 (17.2)	44/192 (23.0)
Right side (%)	31/88 (35.2)	20/46 (43.5)	21/58 (36.2)	72/192 (38.0)
Left Side (%)	34/88 (38.6)	15/46 (32.6)	27/58 (46.6)	76/192 (39.6)
Arm weakness (%)	47/87 (54.0)	25/46 (54.3)	24/53 (45.3)	96/186 (51.6)
Leg weakness (%)	39/85 (45.9)	21/46 (45.7)	20/53 (37.7)	80/184 (43.5)
Facial weakness (%)	43/81 (53.1)	27/46 (58.7)	23/53 (43.4)	93/180 (52.0)
Speech Problems (%)	50/76 (65.8)	29/42 (69.0)	23/50 (46.0)	102/168 (60.7)
Conscious level				
Alert (%)	74/88 (84.1)	42/48 (87.5)	57/59 (96.6)	173/195 (89.0)
Drowsy (%)	9/88 (10.2)	5/48 (10.4)	2/59 (3.4)	16/195 (8.2)
Stupor (%)	3/88 (3.4)	0/48 (0)	0/59 (0)	3/195 (2.0)
Coma (%)	2/88 (2.3)	1/48 (2.1)	0/59 (0)	3/195 (2.0)

For the patients with a final diagnosis of stroke, the proportions dispatched as ‘stroke’ or ‘not stroke’ (dispatch accuracy) for each of the three periods, and for each of the 26 observations weeks are given in Table 4.

**Table 4:** For each observation week and period of study, number of patients with a final diagnosis of stroke and number dispatched as stroke

**Period of study**	**Week**	**Observation**	**Number with final diagnosis of stroke**	**Number dispatched as stroke**	**Percentage dispatched as stroke**
Pre	1	1	16	12	75%
Pre	4	2	11	7	64%
Pre	7	3	11	5	45%
Pre	10	4	10	6	60%
Pre	13	5	17	10	59%
Pre	16	6	3	3	100%
Pre	19	7	8	6	75%
Pre	22	8	11	6	55%
Pre	25	9	5	3	60%
Pre	TOTAL		92	58	63%
During	28	10	3	1	33%
During	31	11	8	6	75%
During	34	12	7	7	100%
During	37	13	10	10	100%
During	40	14	7	6	86%
During	43	15	8	8	100%
During	46	16	5	4	80%
During	TOTAL		48	42	88%
Post	49	17	3	2	67%
Post	52	18	4	4	100%
Post	55	19	6	4	67%
Post	58	20	5	4	80%
Post	61	21	8	8	100%
Post	64	22	7	5	71%
Post	67	23	4	4	100%
Post	70	24	8	6	75%
Post	73	25	7	6	86%
Post	76	26	7	4	57%
Post	TOTAL		59	47	80%
Overall			199	147	74%

**Figure 1:**
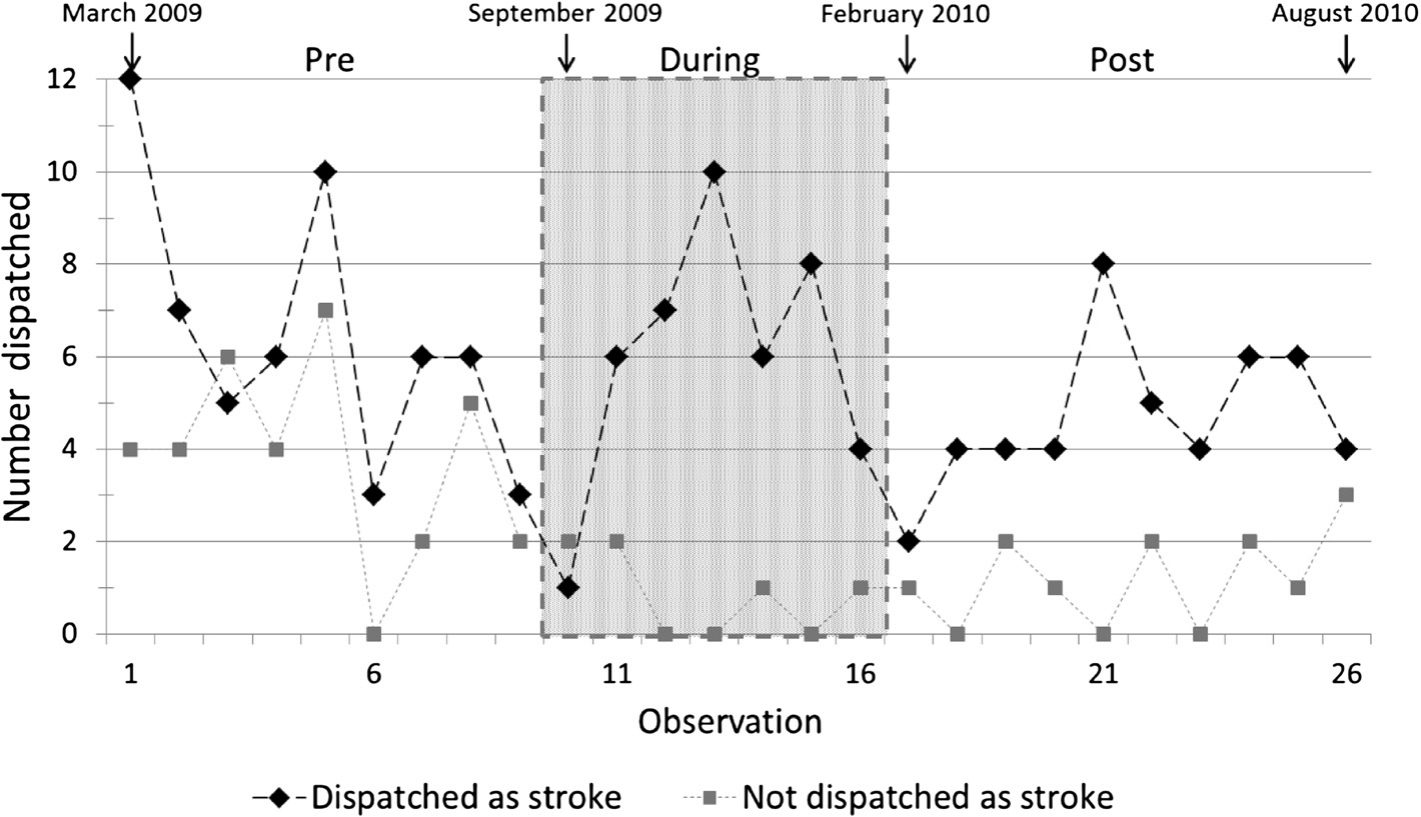
Weekly numbers of patients with final diagnosis of stroke dispatched as stroke and not stroke.

**Figure 2:**
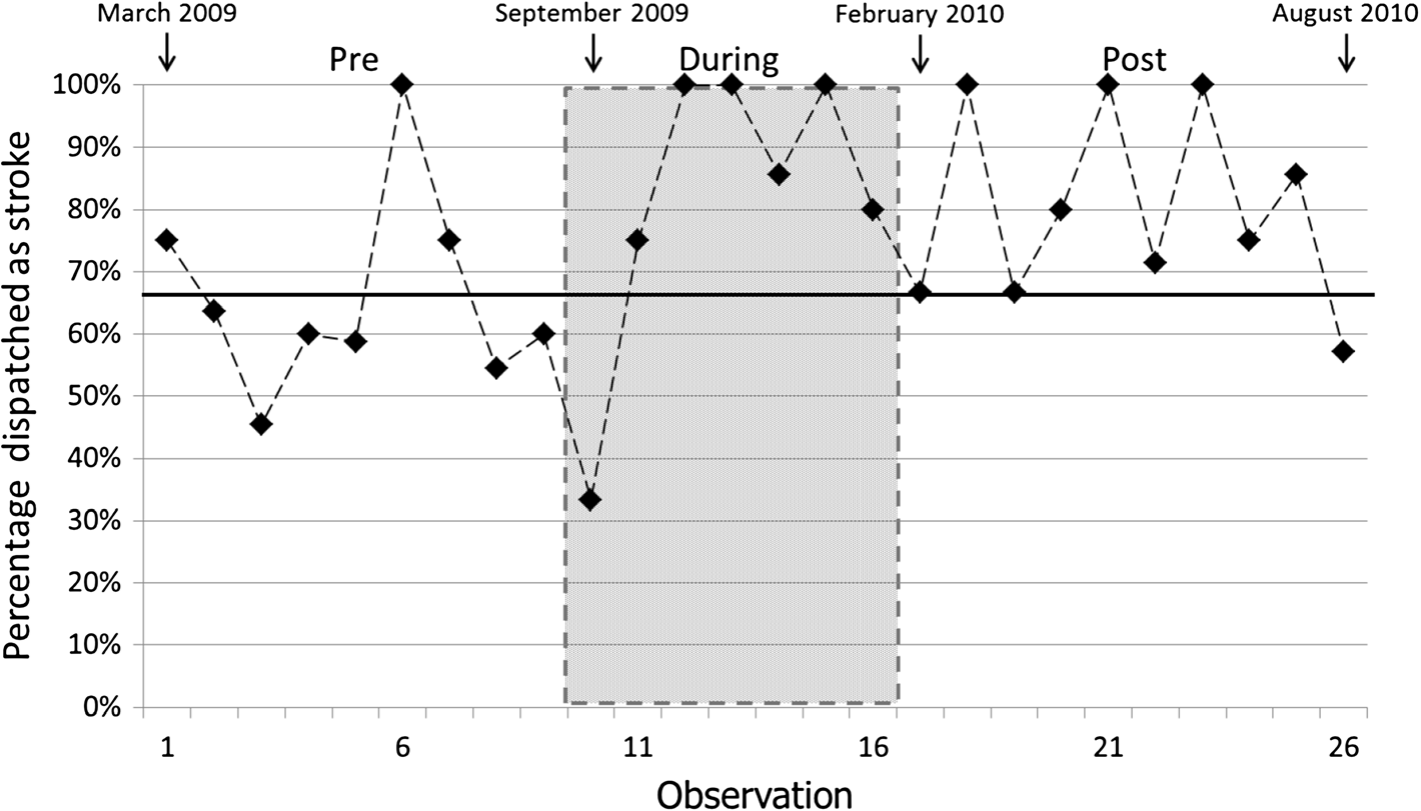
Weekly percentages of patients dispatched as stroke (denominators are patients with a final diagnosis of stroke).

Trends in the number dispatched as stroke and the number dispatched as not stroke are illustrated in Figure 1 and the corresponding diagnostic accuracy is shown in Figure 2. Logistic regression showed no significant change in trend in diagnostic accuracy across the observation weeks (p=0.18), nor an overall trend in diagnostic accuracy across the observation weeks (p=0.85). However, a significant change in dispatch diagnosis accuracy between periods was detected (p=0.003), reflecting an improvement in dispatch accuracy relative to the Pre-implementation period both the During-implementation (OR=4.10 [95% CI 1.58 to 10.66]) and Post-implementation (OR=2.30 [95% CI 1.07 to 4.92]) periods; the difference in dispatch accuracy between During- and Post-implementation periods was not significant (p=0.29). There was no evidence of (first-order) autocorrelation of residuals (r=−0.0123, p=0.95), so no adjustment of standard errors was necessary. These findings were robust to the various imputations applied: the difference between periods in proportions correctly dispatched as stroke was significant for all imputations, with p-values ranging from 0.001 to 0.017. When data from the During- and Post-implementation periods were combined and compared with the Pre-implementation data the overall effect of training was significant (p=0.002; OR=2.90 [95% CI 1.50 to 5.61]).

For the 199 patients with a final diagnosis of stroke and dispatch data, time of call was missing for three (1.5%). Of the remainder, 25 had missing arrival at hospital time. Twenty-two of these had admission to hospital time recorded and one further patient had triage time recorded, so these were used for the time of arrival at hospital. One hundred and ninety four patients had mean (SD) call to arrival at scene 13.1 (12.0) minutes [median 9, IQR 7–15, range 3–99 minutes] and mean (SD) call to arrival at hospital 47.0 (16.3) [median 44, IQR 36–53, range 22–124 minutes].

**Figure 3:**
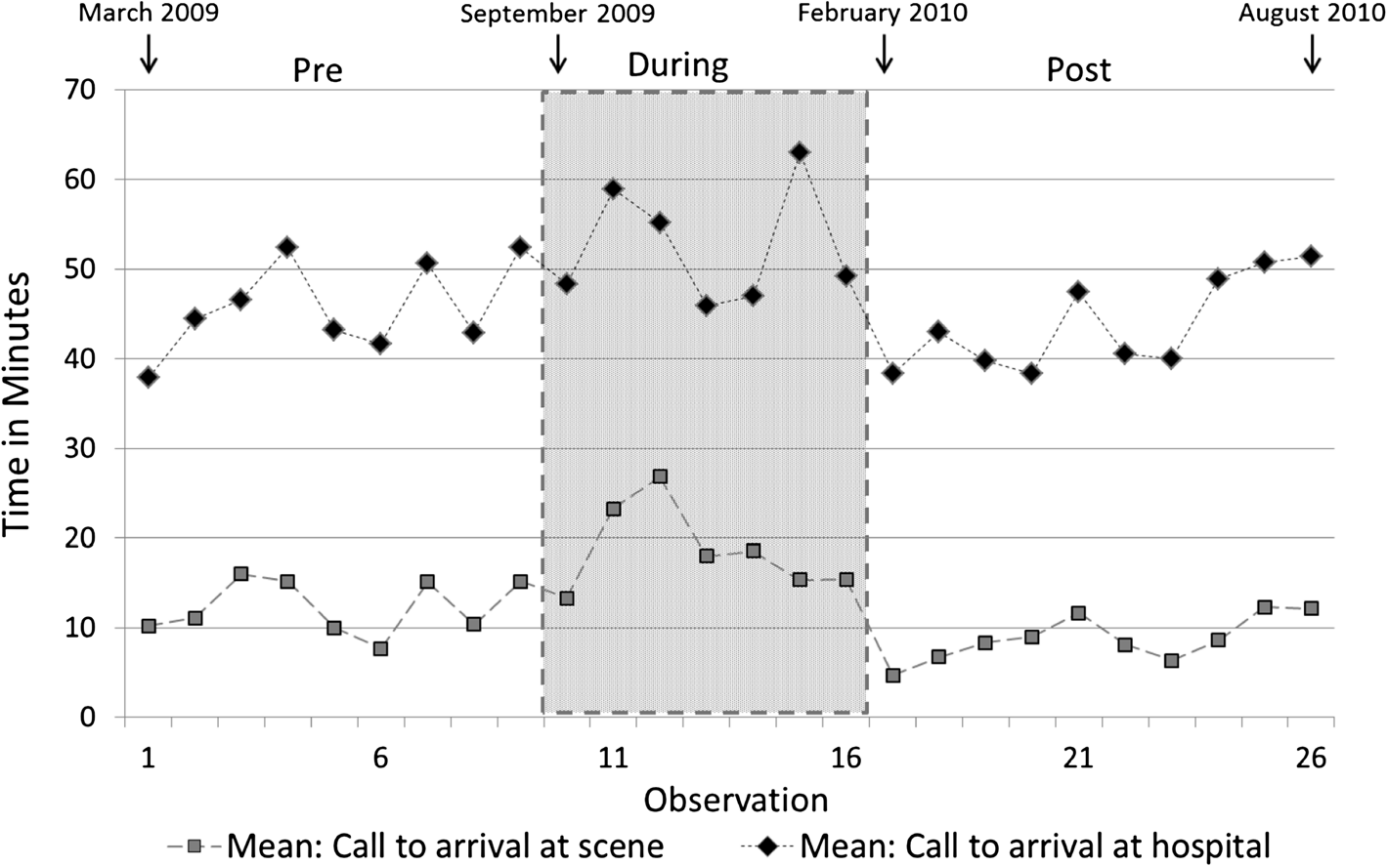
Weekly mean times from call to arrival at scene and times from call to arrival at hospital.

There was a marginally non-significant reduction in mean time from the EMS call to arrival of the ambulance at the scene of 2.8 minutes (95% CI −0.2 to 5.9 minutes, p=0.068) between Pre- and Post-Implementation periods (Table 5). However, these mean times increased significantly overall During-implementation (7.0 minutes; 95% CI 3.8 to 10.3 minutes, p<0.001). There was no evidence of autocorrelation of residuals (r=−0.16; p=0.40), so adjustment of standard errors was not deemed necessary. Overall, there was no significant underlying trend in mean time from the EMS call to arrival of the ambulance at the scene (p=0.18) but potentially appeared to decline over the During-implementation period (p=0.081). Findings were similar when geometric rather than arithmetic means were modelled. Figure 3 shows the weekly mean times from call to arrival at scene and times from call to arrival at hospital.

**Table 5:** Time in minutes from call to: arrival at scene; arrival at hospital; for patients with a final diagnosis of stroke and dispatch data

**Implementation period (n)**	**Mean call to arrival at scene (SD)**	**Median call to arrival at scene (IQR)**	**Mean call to arrival at hospital (SD)**	**Median call to arrival at hospital (IQR)**
Pre (88)	12.2 (8.0)	10 (8–14.8)	45.0 (13.5)	43.5 (37–50)
During (48)	19.2 (19.3)	12.5 (7–21.8)	52.9 (22.7)	48.0 (35.3-61.5)
Post (58)	9.4 (6.2)	8 (6–10.3)	44.9 (12.5)	43 (36.0-52.0)

The mean call to arrival at hospital time was unchanged between Pre- and Post-implementation periods (difference −0.1 minutes; 95% CI −5.5 to 5.3 minutes, p=0.23). However, there was a significant short-term increase During-Implementation of 7.9 minutes (95% CI 2.2 to 13.6 minutes, p=0.009). There was no evidence of autocorrelation of residuals (r=−0.26, p=0.16), so adjustment of standard errors was not deemed necessary. Overall, there was a significant underlying trend in mean time from the EMS call to arrival of the ambulance at the scene (p=0.028) but no evidence that this underlying trend varied between periods (p=0.55). Findings were similar when geometric rather than arithmetic means were modelled.

## Discussion

Implementation of stroke-specific on-line training resulted in an increased recognition of stroke by EMDs and at most a modest reduction in the time from the call to the ambulance’s arrival at the scene.

Prior to training, for every 10 stroke calls made, approximately 6 were correctly identified by the EMDs. This increased to 8 out of 10 after training was implemented and was just short of 9 out of 10 during implementation. The recognition of stroke even at baseline was much higher in comparison to previous reports in the literature, where studies have shown that EMD sensitivity for identifying stroke is below 50% [3,5,6]. One explanation for this may be the Face Arm Speech Time to dial 999 (FAST) mass multi-media public awareness campaign [14], which was coming to an end when Phase 8 started; may have raised awareness of stroke in both the public and EMDs. Despite the relatively high proportion correctly recognised as stroke by EMDs at baseline, the difference between periods in proportions of strokes, correctly dispatched as such, was both clinically and statistically significant. This effect was robust to assumptions made about missing dispatch and final diagnosis, and there was no evidence of non-independence. The difference between Pre- and During-implementation in the proportions of strokes being dispatched as such was seen as a step change close to the time that the training package was introduced, rather than a trend, evidence of which would have been visible through the use of an interrupted time series design. This step change strengthens the evidence for the effectiveness of the intervention because it suggests that the cause of the change happened close to the time of the introduction of the training, making it less plausible that it was due to an external influence. These proportions were similar or better During-implementation than Post-implementation. This slight reduction between During- and Post-implementation may indicate that the effect of the training had reduced slightly, but the difference between Pre- and Post-implementation was still significant, indicating that within the six months of Post-implementation the training was still having a beneficial effect on the EMDs. Nevertheless, future studies should consider exploring the longer term impact of training.

We believe that this is the first study to evaluate stroke training for EMDs and subsequently demonstrate an improvement in the recognition of stroke by EMDs. To our knowledge, no previous studies have evaluated EMD training in relation to the recognition of stroke; therefore, this is the first study to improve the recognition of stroke by EMDs. Previous studies have evaluated educational programmes aimed at improving paramedic, hospital and community awareness [15]. This increased paramedic diagnosis of stroke from 61% to 79% [15] and demonstrated that stroke specific training for EMS personnel can be effective in improving stroke recognition. This suggests that there is a potential for increasing pre-hospital recognition of stroke further by including training for ambulance staff. In a previous study we have shown that on-line learning for EMS staff can increase stroke knowledge and provides the opportunity for continuing professional development [16], but in that study there was no assessment of diagnostic accuracy or timeliness of service. The training package also appeared to reduce pre-hospital delay in terms of call to arrival at scene by a small amount, although a statistical significant reduction was attained only during rather than post the implementation; however, no corresponding reduction was observed in the time interval between call and arrival at hospital. One previous study, (that involved paramedic training including implementation of National Stroke Guidelines, the use of the Los Angeles Pre-hospital Stroke Scale, and a mass media campaign) reported that following the implementation of the package, the time from dispatch to arrival at hospital increased from 42.2 minutes to 45.8 minutes [15]. Our study shows that EMD training not only improves recognition of stroke but may have the potential to contribute toward reducing pre-hospital delays, at least in terms of call to arrival at scene. There are potentially many factors that can influence the time between a call being made and a patient arriving at hospital, from the dispatch code and priority through to road conditions. An improvement in the correct identification of stroke patients has the greatest probability of influencing the time between call and arrival at scene through ensuring the correct dispatch code, vehicle, and priority are used. Our findings are consistent with such an effect. A recent study has reported that there was a significant increase in thrombolysis frequency and a shorter time to the stroke unit for patients who were given the highest dispatch priority (immediate ambulance), compared to an ambulance dispatch time of within 30 minutes [17]. This emphasises the importance of correctly identifying stroke patients, who can then be dispatched with a high level of priority, Category A in the UK.

The reason that the effect on time from call to arrival at scene was not reflected in a reduced time from call to arrival at hospital is unclear. Also unclear is the reason for the pattern of times During-implementation, in particular the number of sharp increases and decreases. Potential explanations outside of the influence of the EMS include presence of road works, shorter daylight hours and the recording of data in December and early January. It is difficult to provide explanations for these patterns, but sampling over longer time frames, to include the same annual periods, and/or increasing the frequency of observations number may have helped with interpreting the data.

### Limitations

The nature of the intervention meant it could not be evaluated as an individually-randomised controlled trial in a single centre and so we used an interrupted time-series design. While this design is not as robust as randomised controlled trial, it is the most robust of quasi-experimental designs, including a simple before and after study [13]. The intervention could have been evaluated using a stronger research design such as a cluster RCT, or even a stepped-wedge cluster RCT, but this would have meant a much larger scale study which would have been beyond the limit of the resources available. However, we recognise that this study provides only preliminary evidence of effectiveness of the training package and further research of its effectiveness using a stronger research design on a larger scale is merited. Moreover, the focus of this study was to evaluate the impact of a training package on the recognition of stroke by EMDs and on the time between the call to the EMS and arrival at hospital rather than evaluating the impact on clinical outcomes. Further research should also explore any impact that the training may have on other outcomes such as time to CT, thrombolysis rates and survival.

We tried to ensure that data capture was over a long period in order to obtain a large enough sample to demonstrate the impact of the intervention. By sampling over 18 months we can be reasonably certain the sample was representative of the annual intake of stroke patients at the centre, and that there was unlikely to be an impact of seasonal variation. In contrast, an 18-months period was a relatively short time series and so has limited the potential for investigation and explanation of underlying trends in the data, for example, in the time from call to arrival at scene and hospital. If resources had allowed we could have increased the time periods over which the data were sampled, which would have allowed further exploration of trends; alternatively, we could have increased the sample size within each observation by sampling for more than seven days. Either or both of these approaches would have increased the numbers and allowed for greater variability in the data. However, this does not invalidate the findings of improved recognition of stroke by the EMDs and the modest reduction in times from Pre- to Post-implementation; effects were estimated with relatively good precision, evinced by the width of the confidence intervals. The accuracy of the recognition of stroke by EMDs was evaluated over seven months in the Post-implementation period; any sustained changes in the level of stroke recognition beyond this time point are not known.

There were differences in the average number of patients identified in the observation weeks between periods. In the Pre-implementation period there was an average of 10 patients per observation week, but in the subsequent During- and Post-implementation periods there were on average approximately 7 and 6 six patients per week. Prior to the beginning of the study the national FAST mass media campaign [14] had just ended. This may explain the larger numbers of patients accessing hospital via the EMS in the Pre-implementation period. As the impact of the FAST campaign dwindled, more patients in the During- and Post-implementation periods may have accessed emergency health care by other means such as direct attendance at the ED and therefore would not have been included in our study. However, this difference in numbers is a reflection of how people access health care rather than how they are dispatched; this would therefore not have impacted on the performance of the dispatchers in correctly dispatching stroke patients as such. It is also highly unlikely that the FAST campaign had an impact on our overall findings: the FAST campaign had ended 6 and 12 months prior to the During- and Post-implementation periods respectively, and so was unlikely to have had an influence on the study conclusions. Supporting this assertion is the fact that whilst there was a significant difference in the number of stroke patients correctly dispatched as such between periods, there was no significant overall trend in observation week, suggesting that there were no strong external influences on correct dispatch rates over the period of the study.

The intervention was implemented in one ambulance service, which may limit generalisability. Also, the EMD recognition of stroke at baseline was high compared with other studies, so it could be argued that the EMDs prior awareness of stroke facilitated further learning. The sample was identified through a retrospective chart review of hospital and EMS records, meaning that the data collected for the study was dependent on previously documented information, and so it was not possible to verify the information through independent assessment. Given the nature of the data that were used, this is unlikely to have affected the results of the study: dispatch data are an objective measure of the code used by EMDs; the stroke register is comprehensive, has been in place over 17 years and is managed by an experienced team.

## Conclusions

This is the first study to develop, implement and evaluate the impact of a training package for EMDs with the aim of improving the recognition of stroke. The findings suggest that in addition to improving the recognition of stroke by EMDs, the training may have the potential to contribute to a reduction in pre-hospital delays. Future research should explore any impact that the training may have on pre-hospital processes and clinical outcomes.

## Competing interests

GF has received honoraria for consultancy and educational activities (Lundbeck; Boehringer Ingleheim). TQ is a member of the steering group for STREAM Trial (Boehringer Ingelheim; Medicines Company for EUROMAX trial) and is a local collaborator/principal investigator for ATLANTIC trial (Astra Zeneca).

## Authors’ contributions

All authors participated substantially in the design and conduct of this study, contributed to data interpretation, take responsibility for the accuracy of the work, and have seen and approved the final version of the article. All authors conceived and designed the study. SPJ acquired the data. CJS provided statistical advice. MJL, SPJ and CJS performed the data analysis. All authors drafted the article, and all authors contributed substantially to its revision. CLW takes responsibility for the paper as a whole. All authors read and approved the final manuscript.

## Pre-publication history

The pre-publication history for this paper can be accessed here:

http://www.biomedcentral.com/1472-6963/13/318/prepub

## References

[B1] Lopez AD Mathers CD Ezzati M Jamison DT Murray CJL Global and regional burden of disease and risk factors, 2001: systematic analysis of population health data Lancet 2006 367 9524 1747 1757 10.1016/S0140-6736(06)68770-9 16731270

[B2] European Stroke Organization (ESO) Executive Committee and the ESO Writing Committee Guidelines for Management of Ischaemic Stroke and Transient Ischaemic Attack 2008 http://www.eso-stroke.org/pdf/ESO08_Guidelines_Original_english.pdf 10.1159/000131083 18477843

[B3] Ramanujam P Guluma KZ Castillo EM Chacon M Jensen MB Patel E Linnick W Dunford JV Accuracy of stroke recognition by emergency medical dispatchers and paramedics –San Diego experience Prehosp Emerg Care 2008 12 3 307 313 10.1080/10903120802099526 18584497

[B4] The National Institute of Neurological Disorders and Stroke rt-PA Stroke Study Group Tissue plasminogen activator for acute ischemic stroke N Engl J Med 1995 333 24 1581 1587 7477192 10.1056/NEJM199512143332401

[B5] Buck BH Starkman S Eckstein M Kidwell CS Hained J Huang R Colby D Saver JL Dispatcher recognition of stroke using the national academy medical priority dispatch system Stroke 2009 40 2027 2030 10.1161/STROKEAHA.108.545574 19390065 PMC2711028

[B6] Deakin CD Alasaad M King P Thompson F Is ambulance telephone triage using advanced medical priority dispatch protocols able to identify patients with acute stroke correctly? EMJ 2009 26 6 442 445 10.1136/emj.2008.059733 19465622

[B7] Kothari R Jauch E Broderick J Brott T Sauerbeck L Khoury J Liu T Acute stroke: delays to presentation and emergency department evaluation Ann Emerg Med 1999 33 1 3 8 10.1016/S0196-0644(99)70431-2 9867880

[B8] Kothari R Sauerbeck L Jauch E Broderick J Brott T Khoury J Liu T Patients awareness of stroke, signs, symptoms and risk factors Stroke 1997 28 1871 1875 10.1161/01.STR.28.10.1871 9341687

[B9] Jones SP Ford GA Gibson JME McAdam JJ Dickinson H McLoughlin A Leathley MJ Quinn T Watkins SL on behalf of the ESCORTT Group Callers' experiences of making 999 emergency calls at the onset of acute stroke: Qualitative study Emerg Med J 2011 10.1136/emj.2010.108563 21742747

[B10] Jones SP Carter B Ford GA Gibson JME Leathley MJ McAdam JJ O'Donnell M Punekar S Quinn T Watkins CL on behalf of the ESCORTT group The identification of acute stroke: an analysis of emergency calls Int J Stroke 2012 10.1111/j.1747-4949.2011.00749 22335960

[B11] Gilbert EH Lowenstein SR Koziol-McLain J Barta DC Steiner J Chart reviews in emergency medicine research: What are the methods? Ann Emerg Med 1996 27 3 305 308 10.1016/S0196-0644(96)70264-0 8599488

[B12] Worster A Bledsoe RD Cleve P Fernandes CM Upadhye S Eva K Reassessing the methods of medical record review studies in emergency medicine research Ann Emerg Med 2005 45 4 448 451 10.1016/j.annemergmed.2004.11.021 15795729

[B13] Wagner AK Soumerai SB Zhang F Ross-Degnan D Segmented regression analysis of interrupted time series studies in medication use research J Clin Pharm Ther 2002 27 4 299 309 10.1046/j.1365-2710.2002.00430.x 12174032

[B14] Department of Health Stroke: Act F.A.S.T. awareness campaign 2011 Dh.gov.uk, 2011 [Internet]. http://webarchive.nationalarchives.gov.uk/20130107105354/http://www.dh.gov.uk/en/Publicationsandstatistics/Publications/PublicationsPolicyAndGuidance/DH_094239 [cited 2011 9^th^ December]

[B15] Wojner-Alexandrov AW Alexandrov AV Rodriguez D Persse D Grotta JC Houston paramedic and emergency stroke treatment and outcomes study (HoPSTO) Stroke 2005 36 1512 1518 10.1161/01.STR.0000170700.45340.39 15961712

[B16] Jones SP McLoughlin ASR Watkins CL Acute stroke management: an online course J Paramed Pract 2011 3 322 327

[B17] Berglund A Svensson L Cjöstrand C von Arbin M von Euler M Wahlgren N Engerström L Höjeberg B Käll TB Mjörnheim S Engqvist A Higher prehospital priority level of stroke improves thrombolysis frequency and time to stroke unit: the hyper acute stroke alarm (HASTA) study Stroke 2012 43 2666 2670 10.1161/STROKEAHA.112.652644 22879096

